# Complete Genome Sequence of a Genotype 3 Atypical Porcine Pestivirus Strain (OKN/2021) from Okinawa Prefecture, Japan

**DOI:** 10.1128/mra.00614-22

**Published:** 2022-11-14

**Authors:** Keigo Ikeda, Kohtaro Miyazawa, Michihiro Takagi, Hisayuki Tomochi, Keiko Ishii, Miho Araki, Yoshifumi Iwamaru

**Affiliations:** a Division of Infectious Animal Diseases, National Institute of Animal Health, National Agriculture and Food Research Organization, Ibaraki, Japan; b Division of Zoonosis Research, National Institute of Animal Health, National Agriculture and Food Research Organization, Ibaraki, Japan; c Okinawa Prefectural Institute of Animal Health, Okinawa, Japan; d Livestock Hygiene Service Center of Okinawa Prefecture, Okinawa, Japan; DOE Joint Genome Institute

## Abstract

We report the complete genome sequence of atypical porcine pestivirus (APPV) OKN/2021, which was sampled in the Okinawa Prefecture, Japan. The sequence bears the closest resemblance to another previously detected Japanese genotype 3 APPV sequence. This genome sequencing will help researchers in Japan learn more about the virus epidemiology and evolutionary characteristics.

## ANNOUNCEMENT

Atypical porcine pestivirus (APPV) belongs to the genus *Pestivirus* and the family *Flaviviridae*. APPV was originally discovered in the United States in 2015 (1), and it has since been associated with congenital tremor (CT) type A-II (2–5). APPV has been found in North America, South America, Europe, and Asia thus far (6). Furthermore, it has been classified into three genotypes, i.e., genotype 1, genotype 2, and genotype 3 (7). APPV strains classified as genotype 3 were mostly detected in China (7). Kasahara-Kamiie et al. recently reported a novel APPV strain belonging to genotype 3, speculating that it might have evolved uniquely in the Japanese pig population (7). The complete genome sequence of another APPV strain, known as OKN/2021, which was derived from the serum of a sucking piglet with CT collected in Nanjo City, Okinawa Prefecture, Japan, in February 2021, is presented here.

The serum RNA was extracted using the High Pure viral RNA kit (Qiagen, Venlo, Netherlands), followed by first-strand cDNA synthesis with the Transcriptor first-strand cDNA synthesis kit (Roche Diagnostics, Basel, Switzerland) to determine the entire genome sequence. Following that, 16 overlapping fragments were amplified using PCR and 31 primers (Table 1). Rapid amplification of cDNA ends (RACE) was used to amplify the 5′ untranslated region (UTR) and the 3′ UTR using a 5′/3′ RACE kit, second generation (Roche Diagnostics), and five specific primers, as shown in Table 1. Sanger sequencing of the amplified products was performed using an ABI 3500 system (Thermo Fisher Scientific, Waltham, MA), and the data were assembled using GENETYX v.15 software (Genetyx, Tokyo, Japan) to determine the whole-genome sequence of the OKN/2021 strain. Phylogenetic analysis of 62 full genomes, including strain OKN/2021, was performed with Molecular Evolutionary Genetics Analysis (MEGA) v.11 as described previously (7).

**TABLE 1:** APPV-specific primers used in this research

Forward primer		Reverse primer		
Primer name	Primer sequence (5′ to 3′)	Primer name	Primer sequence (5′ to 3′)	Reference
		5′RACE_168R	CCGTACTCGGGGCTTAAGAGTT	7
		5′RACE_278R	CCAGGTCCCCCACCGATTTCT	7
		5′RACE_363R	GATTTTATACTCTTACCTGAGGCAT	7
1F	TGCATAATGCAAAAATTGGCTGCA	1431R	GACTAGTRTCTATCATTCCCAA	8
682F	GGAAATTTAGTGGCCCTCCT	878R	CTTCCATTGGTAAAACCCCC	
1257F	AAAGTYCAATGGTTTTTGAAGG	3370R	AGRAAGCTCAAGGCTACTGGGC	8
1903F	ACACCGTAACGGGGATGTAT	2752R	TCAAGGACATTATCGCCAGG	
3238F	TATGGAAGATGGATTGGACGGA	5139R	ACCTCATRAAGCGRCATACACT	8
3890F	CCTCCTATATGCGGAAACCA	4554R	TTTTCTCACCAGTGACGTGC	
4968F	GAGTTGGCAAGCATGCGGAGA	6750R	CCTGATGYTTCTTCAAGTAYTG	8
5569F	CAGAGGCGGAAGCTAAAGAA	6125R	CGGATGGGTACTTCTTTCCA	
6600F	GACCAYCAGCTGAGACGATTGC	8226R	TTTTGGACCCTCGRTGTGCTTT	8
7194F	AACAGGAGTCTTGTCACGCA	7412R	TTCCGGCTCCAGTTTGTCTA	
7281F	GGGCTTGGATTATGTGTGCT	8280R	TAATTTCCGACCCCAAGTCG	
8101F	GCCAGGTAGCCATAGGYAAAGT	10122R	ATTGGGCCCAGTCTGCACTGAC	8
8680F	CCTCACCCCCAAACATAAGA	9493R	CTCCCATTTACTTTGCCGAG	
9961F	AGAAACCGCGAGTGATACAGT	11458R	CAAGTATTTACAACAACCCCAC	8
		11567R	GGGGCCTCCTTGCTTCATCTAGAT	8
10531F	CTTTTGGCAAGCCTCAGAAG	10927R	ACGTCCTCCAATTTATGCCC	
3′RACE_10997F	AAAGACGAGCCAGCGGTTAGTCGA			8
3′RACE_11247F	AAAATGAAGCTCATGAAGAAGATG			

**FIG 1:**
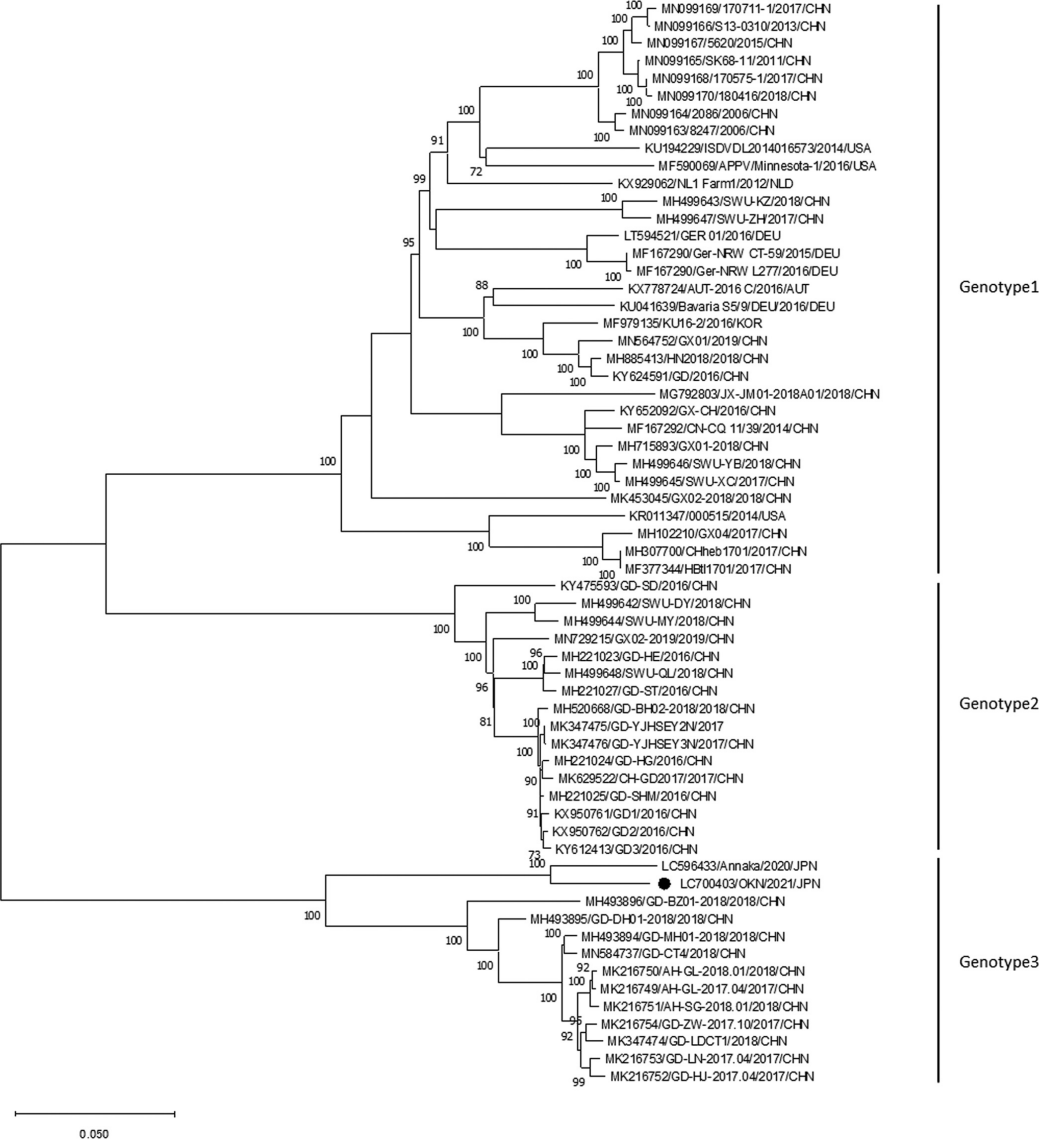
Phylogenetic tree of APPV strains based on the complete sequences. The phylogenetic tree was generated by the maximum likelihood method using MEGA v.11 with 1,000 bootstrap replicates (values of <70 are omitted). OKN/2021 is marked with a black circle.

The complete genome sequence of the OKN/2021 strain was 11,567 bp in length, with a GC content of 46.3%. The sequence was composed of a 5′ UTR of 377 nucleotides (nt), a single large open reading frame (ORF) of 10,908 nt, and a 3′ UTR of 267 nt. The ORF encoded a predicted polyprotein of 3,625 amino acids. The OKN/2021 strain belongs to genotype 3, with the closest relationship to the Anna/2020 strain (GenBank accession number LC596433) identified in Japan, according to phylogenetic analysis. These two strains were in the same branch of the phylogenetic tree, and the other strains identified in China formed a different branch in genotype 3 (Fig. 1). The OKN/2021 strain displayed 94.4% nucleotide identity and 97.1% amino acid identity to the Anna/2020 strain.

In this study, we identified the complete genome sequence of the OKN/2021 strain. This strain showed the greatest genetic similarity to the Anna/2020 strain and formed a branch independent from Chinese genotype 3 strains, implying that endemic APPV strains might exist in the Japanese pig population. To better understand the distribution of APPV genotypes in Japan, further research is needed.

### Data availability.

The APPV OKN/2021 sequence is available in GenBank under accession number LC700403. The raw reads have been deposited in the Sequence Read Archive (SRA) under BioProject accession number PRJDB14424.
